# Gene Therapy Approaches to Biological Pacemakers

**DOI:** 10.3390/jcdd5040050

**Published:** 2018-10-19

**Authors:** Melad Farraha, Saurabh Kumar, James Chong, Hee Cheol Cho, Eddy Kizana

**Affiliations:** 1 Centre for Heart Research, the Westmead Institute for Medical Research, The University of Sydney, Sydney, NSW 2145, Australia; mfar4366@uni.sydney.edu.au (M.F.); james.chong@sydney.edu.au (J.C.); 2 Sydney Medical School, The University of Sydney, Sydney, NSW 2006, Australia; 3 Department of Cardiology, Westmead Hospital, Westmead, NSW 2145, Australia; Saurabh.Kumar@health.nsw.gov.au; 4 Departments of Pediatrics and Biomedical Engineering, Emory University, Atlanta, GA 30322, USA; heecheol.cho@emory.edu

**Keywords:** atrioventricular node, bradycardia, gene therapy, heart, pacemaker, sinoatrial node, viral vector

## Abstract

Bradycardia arising from pacemaker dysfunction can be debilitating and life threatening. Electronic pacemakers serve as effective treatment options for pacemaker dysfunction. They however present their own limitations and complications. This has motivated research into discovering more effective and innovative ways to treat pacemaker dysfunction. Gene therapy is being explored for its potential to treat various cardiac conditions including cardiac arrhythmias. Gene transfer vectors with increasing transduction efficiency and biosafety have been developed and trialed for cardiovascular disease treatment. With an improved understanding of the molecular mechanisms driving pacemaker development, several gene therapy targets have been identified to generate the phenotypic changes required to correct pacemaker dysfunction. This review will discuss the gene therapy vectors in use today along with methods for their delivery. Furthermore, it will evaluate several gene therapy strategies attempting to restore biological pacing, having the potential to emerge as viable therapies for pacemaker dysfunction.

## 1. Introduction

The sinoatrial node (SAN) is a group of highly specialized cells, containing less than 10,000 genuine pacemaker cells, keeping the mammalian heart beating regularly [1,2]. The SAN however, can become defective via several means including: myocardial infarction, cardiomyopathy, genetic defects but most prevalently because of ageing [3,4,5]. This condition affects approximately 1 in 600 cardiac patients older than 65 years and accounts for 50 percent or more of permanent pacemaker insertions in the United States alone [4,6], with the incidence rising due to the world’s ageing population [7]. Dysfunction of the SAN leads to heart rate control issues including bradycardia, with severe cases resulting in sudden cardiac death [8,9]. To date, there are no known cures for SAN dysfunction, with the only viable management option being the insertion of an electronic pacemaker. 

Implantable electronic pacemaker technology has continued to evolve since its development six decades ago [10,11]. Today’s modern devices can sense the intrinsic rhythm in both the atrium and ventricle and can pace either chamber on demand at programmable baseline rates [11,12]. Furthermore, advancements in battery technology and software algorithms allows devices to be powered for longer periods of time [12,13,14,15]. Although effective, these devices and their subsequent insertions present their own complications. Myocardial perforations can occur during pacemaker deployment, occurring in up to 1% of insertion cases [11,16,17]. Patients can develop a pneumothorax, wound hematomas or venous thrombosis. Follow up surgeries are needed to correct pacemaker lead failure. These are electronic devices and as such, battery changes are required as they diminish. Most seriously, hardware related infections can manifest, with patients requiring effective antibiosis to be established before a replacement device can be re-inserted [11,13].

The complications associated with electronic pacemakers and the existence of a need for better treatment of SAN dysfunction has motivated research into discovering more effective and innovative treatment options. Advances in cellular and molecular biology over the past two decades have spawned exciting avenues which show potential in addressing the limitations of current treatment options. With recent improvements in stem cell development, gene transfer vectors, delivery methods, and viable options for clinical translation, several inroads have been established for treatment of cardiovascular diseases. In this review, we discuss the current experimental approaches using gene therapy for the development of biological pacemakers, and the therapeutic prospects of gene therapy for addressing SAN dysfunction in humans.

## 2. Prerequisites for the Generation of a Biological Pacemaker

The SAN can generate electrical impulses faster than those generated in other cells around the heart. It spontaneously depolarizes during diastole to initiate the subsequent heartbeat. The I_f_ (funny) current generated by these nodal cells flows through hyperpolarization-activated cyclic nucleotide-gated (HCN) channels, which are cation channels activated by hyperpolarization. This current is mainly involved in diastolic depolarization, responsible for keeping the heart beating regularly [18,19,20,21]. The other important current is the inward rectifier potassium (Kir) channel current (I_K1_) flowing through Kir channels. These channels close upon depolarization, suppressing membrane repolarization helping to maintain more prolonged cardiac action potentials and a quiescent state [22].

HCN4 is a channel isoform highly expressed in the SAN. HCN4 mutations have been shown to cause sinus node dysfunction [23,24,25]. Overexpressing HCN4 specifically in the heart or delivering cardiomyocytes overexpressing HCN4 exhibited pacemaker activity in small animal models [26,27]. On the other hand, working cardiomyocytes maintain the resting membrane potentials during diastole. The I_K1_ current flowing through Kir channels plays an important role in this phenomenon. Left ventricular cardiomyocytes of guinea pigs transduced with dominant-negative Kir2.1 showed spontaneous action potentials [22,26]. Additionally, I_K1_-enhanced human-induced pluripotent stem cell-derived cardiomyocytes (hiPSC-CMs) lose spontaneous beating and acquire stable resting membrane potentials [28].

Different biological approaches to enhance cardiac automaticity have been investigated over the years. The common goal is to generate an ectopic region of automaticity in the heart that can function as a surrogate for the SAN. To do this, enhancement of the I_f_ current and/or attenuation of the I_K1_ current are prerequisites for spontaneous diastolic depolarization of SAN cells [29].

The number of cells required to pace the heart however is not as well defined. In the native heart the number of genuine pacemaker cells is estimated to be less than 10,000 [1]. Earlier studies with spontaneous beating embryoid bodies derived from human or mouse embryonic stem cells suggested that comparable number of cells could pace the rodent ventricle upon transplantation [30,31]. It is less straightforward to estimate the number of pacemaker cells needed to achieve sustainable pacing in the case of gene-based biological pacemakers. One would need to estimate the number of de novo, in situ-reprogrammed pacemaker cells by immunostaining and 3-d reconstruction, for example. Furthermore, a dosing study will likely be needed to find the dose that yields sustainable cardiac pacing in an animal model of chronic bradycardia.

## 3. Cell-Based Approaches to Biological Pacemakers

Cell-based approaches to biological pacemakers involve transplanting a cluster of spontaneously beating cells into the heart, to induce pacemaker activity [13,32,33]. The first cell-based system transplanted dissociated fetal canine cardiomyocytes into the myocardium of the left ventricular free wall with AV block. Ectopic ventricular escape rhythms appeared in two implanted animals but not in the controls, with the group suspecting the implanted cells were functionally coupling with the host cardiomyocytes [34]. Although successful, the origin of the cells raised ethical issues, the cells engraftment was poor, and transplantation requires extensive immunosuppression. The cell transplantation approach, from then on, was sporadically mimicked using various spontaneously beating cell types derived from different sources [32,33].

Human embryonic stem cells (hESCs) readily differentiate into spontaneously beating cardiomyocytes and provide a more robust source of pacemaker-like cells [35,36]. In vivo transplantation of hESC-derived cardiomyocytes into guinea pigs resulted in biological pacemaker activity confirmed by ex vivo optical mapping [30]. SAN-like pacemaker cells generated from hESCs by transgene independent differentiation, demonstrated the capacity to function as a biological pacemaker when transplanted into the apex of rat hearts [36]. Furthermore, pigs with chronic AV block, transplanted with spontaneously active embryoid bodies, showed an induction of significant pacemaker function that persisted for weeks [37]. Once again, the downside to this approach was the controversial source of the cells and the need for strong immunosuppression to maintain engraftment of the transplanted donor cells.

An alternative strategy that can potentially overcome the contentiousness and immunological issues present with hESC derived pacemaker cells is to use autologous-induced pluripotent stem cells (iPSC). These cells are created using a cocktail of transcription factors to de-differentiate adult cells obtained from skin or hair, into a pluripotent state [38]. They are then subsequently differentiated into pacemaker-like cells which are then used in in vitro and in vivo experiments [39,40,41]. In one study, iPSC-derived cardiomyocytes were delivered into dog hearts by open thoracotomy. Biological pacemaker activity was seen in only 50% of the animals, with beating rates of 40–50 bpm [42].

Although more attractive, iPSC-derived biological pacemakers still face substantial hurdles. Current iPSC technologies produce mixed population of cells with various phenotypes. One approach to enhance the pacemaker cell population is to overexpress a transcription factor, such as Shox2, which is specific to the sinoatrial node development during differentiation of pluripotent stem cells [31]. It however, may be impractical or perhaps unnecessary to attain pure populations of pacemaker cells for the purpose of biological cardiac pacing. The native SAN is indeed a highly heterogeneous tissue, in which atrial myocytes, transitional myocytes with both atrial- and nodal-like phenotypes as well as non-myocytes reside [43,44,45].

Safety concerns also still exist around the use of iPSCs due to their relative immaturity [46], potential to migrate or differentiate into other cell types, and tumorigenicity [47]. Antiviral immune responses might be generated against the transcription factors introduced via viral vectors to de-differentiate the primary adult cells. However, this concern is decreasing as non-viral, non-integrating methods become more widely used [48]. Moreover, generating iPSCs requires weeks to months, meaning a patient would have to defer treatment until their cells became available. This limits the potential patient pool to those not requiring urgent chronotropic support.

Therefore, other treatment modalities via gene therapy need to be explored to address the shortcomings of using cell-based approaches to biological pacemakers.

## 4. General Principles of Myocardial Gene Therapy

In the broadest of terms, gene therapy refers to the delivery of nucleic acid sequences to manipulate gene expression in target cells or tissue, with the aim of effecting a specific therapeutic outcome. Three criteria are required for successful gene therapy. (1) The selection of a gene transfer vector. (2) A suitable vector delivery method and (3) A therapeutic gene target. Every application of gene therapy requires all three of these components specifically tailored to the application.

There are three main approaches to gene therapy. The classic approach is the overexpression of a gene of interest. This involves using a constitutively active and/or tissue specific promoter to express a gene that is normally not available or is down-regulated. Overexpression of this gene however is predicted to have a therapeutic effect [2]. The second approach employs regulatory RNA molecules such as siRNAs and miRNAs to target and turn off genes in a sequence dependent manner [49,50]. The final approach and the most contemporary employs molecular techniques to alter the DNA code to repair gene defects [51]. Each of these approaches present their own strengths and limitations, each being selected based on the application required.

### 4.1. Vectors

The most common forms of gene therapy entail the transfer of nucleic acids via a viral vector. Viral vectors are exploited because of their natural ability to efficiently deliver genetic material to cells or tissue. Gene therapy applications use viral genomes which are highly edited, so that they become non-infectious and replication defective. Therefore, posing minimal risk to the host and surrounding environment [52,53,54].

Several viral vectors are currently used for gene transfer applications in a cardiac context. The four most commonly used methods are compared in Table 1. Plasmid DNA is commonly used for in vitro transfection experiments as it is the easiest to produce and has a large packaging capacity. It has been used in cardiac applications. However, its main shortcomings include a very low transfection efficiency in in vivo applications and very transient gene expression [55,56]. This therefore limits its application for pacemaker development. Adenoviral vectors are the most widely used in cardiac gene therapy applications. They can transduce the mammalian heart with a very high efficiency, allowing for robust proof-of-principle studies. This vector’s main limitation is the induction of strong and rapid inflammatory and immune responses, resulting in a limited expression window of two weeks [2,57,58,59,60]. Lentiviral vectors are capable of long-term gene expression via their ability to integrate into the host cell genome. They however are not as cardiotropic, requiring direct injections only. There is also the attendant risk of insertional mutagenesis, because of the integrating nature of the vectors’ biology [61,62,63,64].

Recombinant adeno-associated virus (rAAV) has become the most promising vector for cardiac-based gene therapy. This vector is derived from a parental virus presumed to be non-pathogenic. Packaging with certain capsid subtypes generates vectors which are highly cardiotropic and capable of excellent cardiac transduction. This vector generates minimal immunogenicity and can therefore confer long term gene expression. The two main limitations this vector possesses include a limited packaging capacity and the vectors have severely inhibited functionality in the presence of pre-existing neutralizing antibodies [65,66,67,68].

#### Improving rAAV Delivery Vectors

As outlined in Section 4.1, each delivery vector possesses its own strengths and weaknesses. Since rAAV has become a promising candidate for human clinical trials, efforts are being undertaken to improve its efficiency at transducing target cells and its ability to evade pre-existing neutralizing antibodies. Two current approaches for generating these improved vectors include “rational design” of novel AAV variants [70] or directed evolution to isolate cardiotropic rAAV variants from AAV libraries with diverse capsids, also helping to evade the immune response [71]. Directed evolution has shown more promise with generating functional, novel, capsid variants in models targeting the liver and lungs [72,73] although minimal work has been done on targeting cardiac cells [74].

### 4.2. Vector Delivery to Myocardium

**Figure 1:**
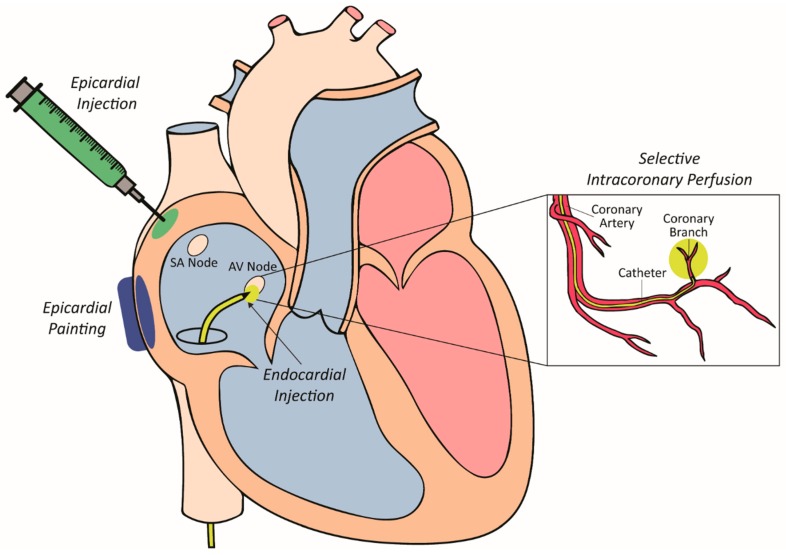
Vector delivery approaches targeting the different sites of the myocardium.

Several approaches for delivering viral vectors in pre-clinical models have been trialed over the past two decades, each presenting their own strengths and weaknesses. These delivery strategies have been developed and optimized for the large mammalian heart as outlined in Figure 1 and lend themselves to clinical translation.

The ideal site of injection in the context of SAN dysfunction would be in the region of the SAN itself. This would ensure synchronous atrial and ventricular activation provided the AV node and the remainder of the conduction system was intact. Injection at other sites within the atrium could also achieve this but perhaps with reduced efficiency due to separation from the native conduction system. Effective, minimally invasive approaches to atrial gene delivery have not yet been developed. The approach used in most of the large animal studies to date to overcome this limitation has been to inject vector into the right ventricle [75,76]. Within this chamber, injection into the septum in the region of the bundle of His allows the biological pacemaker to capture the natural conducting system and avoid ventricular desynchrony. As such, the following sections discuss the most common approaches for delivering viral vectors to these sites.

#### 4.2.1. Epicardial Painting

Epicardial painting was one of the first methods used for targeted vector delivery to the atrium [77,78]. This method, used in pre-clinical pig models involved exposing the atria and painting a mixture of vector, poloxomer and varying concentrations of the proteolytic enzyme trypsin to the epicardial surface. The poloxomer, a synthetic polymer, is liquid at 4 degrees celsius, allowing it to be mixed with the vector and painted onto the atria. Upon warming it becomes solid and helps to immobilise and contain the vector to the epicardial surface of the atria. Furthermore, the trypsin is used to dissolve the epicardial extracellular matrix, allowing the vector to penetrate directly into the atrial muscle. Importantly, atrial muscle weakening and inadvertent transduction of the ventricles did not occur following gene transfer by this method [79]. This approach could therefore be trialled as a delivery method in pacemaker development by painting a small area of myocardium to create a focal point for gene transfer. This approach, however, requires an invasive surgical thoracotomy for access to the epicardial atrial surface.

#### 4.2.2. Epicardial Injection

Direct epicardial injections have also been used for vector delivery [52,80]. This process involves performing a thoracotomy to expose the epicardial surface of the heart. Once exposed, small gauged needles are used to inject directly into the heart wall. This method leads to localised and high-density transgene expression around the needle track. Electroporation has also been used in conjunction with epicardial injections to increase transduction efficiency [81,82]. This dual modality increased transduction efficiency from 10% to 50% in atrial cells [50,81,82]. The major limitation of this approach is the need for invasive surgery via a thoracotomy to expose the epicardial surface of the heart. This increases the risk of major complications during the procedure, and in the postoperative period. There is also the probability of heterogeneous gene expression, injection-related tissue damage triggering an acute inflammatory response [52,83] as well as potential clinical issues such as ventricular fibrillation if the electroporation shocks are not synchronised with ventricular electrical activation [84].

#### 4.2.3. Selective Intracoronary Perfusion

Arterial perfusion was created as a way to selectively deliver a gene therapy vector to a smaller area of myocardium (atrioventricular node) to modify its electrophysiology [85]. This approach involves advancing a perfusion catheter with a small lumen as far down as possible in the target coronary artery. The advantages of this approach include minimal invasiveness, and delivery of vectors can be achieved using standard clinical approaches with off-the-shelf equipment. The main disadvantage of delivery by intracoronary perfusion is the limited efficacy of transduction largely due to the following hurdles: (1) overcoming innate microvascular tone; (2) vascular endothelial barrier to access cellular targets and; (3) local vector–target cell interactions [86,87,88,89,90,91]. To help overcome these barriers nitroglycerin, adenosine, vascular endothelial growth factor and low calcium crystalloid administration have been assessed. High levels of gene transfer have been able to be achieved at the target site of the atrioventricular (AV) node by using an intracoronary perfusion protocol [85]. In a pig model, the right coronary artery was catheterised, and its AV nodal branch cannulated. The latter was infused with a cocktail of agents to dilate and permeablise the vasculature followed by vector delivery. With this approach, a transduction efficiency close to 50% of AV nodal cells was able to be achieved, allowing for electrophysiological modification of AV node function.

#### 4.2.4. Intramyocardial Injection

The intramyocardial injection method presents the most attractive way to deliver vectors to the mammalian heart, especially for biological pacemaker development [75,92]. The process involves the advancement of an endocardial needle catheter, under fluoroscopic guidance to the site of injection. Once in position, the needle is advanced into the myocardial wall to inject the vector directly. Direct intramyocardial injections have several advantages over other methods, especially in the case of biological pacemakers: (1) vectors can be delivered at very high local concentrations, due to minimal leakage into the surrounding tissue, (2) direct injection bypasses the endothelial barrier, which acts as a formidable barrier for efficient gene transfer, (3) off-target organ biodistribution is minimized due to the decreased spread of the vector outside the injection site and (4) the neutralizing effect of pre-existing antibodies is stunted as there is decreased exposure to the humoral immune response. In the case of biological pacemakers using gene therapy, direct intramyocardial injection is the ideal approach as percutaneous, catheter-based injections are minimally invasive and a focal site is desirable for pacing.

## 5. Gene Therapy Approaches to Biological Pacemakers

Different approaches using gene therapy have been investigated over the past two decades, to enhance cardiac automaticity. The goal of all the approaches is to generate a region of automaticity in the heart that can function as a replacement for the SAN. The following section will discuss the various gene-based attempts to generate biological pacemakers.

### 5.1. Receptor-Based Gene Therapy Apporach

The sinus node has a higher density of β adrenergic receptors as compared to the surrounding atrium [93,94]. The increased density of this receptor near the SAN suggested that it regulated the I_f_ current and could therefore result in an increased heart rate [95]. One of the earliest gene therapy approaches for biological pacing looked at increasing the heart rate of mice [93,96] and pigs [97] by overexpressing the human form of the β_2_ adrenergic receptors in the atrium. Although this approach did not generate a so-called biological pacemaker, endogenous SAN rates were accelerated by up to 50% in treated animals as compared to the controls. The limitation of this approach was that the diseased phenotype of pacemaker dysfunction was not addressed and the β_2_ receptors were used in a non-specific manner to stimulate the heart rate. This approach could therefore influence other channels which were thought to be stimulated in a similar manner.

### 5.2. Channel-Based Gene Therapy Approaches

#### 5.2.1. Kir2.1 Channel Downregulation (Kir2.1AAA)

As outlined previously, I_K1_ and other potassium currents contribute to action potential repolarization and establish diastolic resting membrane potentials. Genetic suppression and down regulation of these currents was used in a way to release the “electrical I_K1_ brake” and generate automatic rhythms.

Reduction in the number of functional Kir channels (encoded by the KIR2 gene family) was achieved in the left ventricular myocardium of guinea pig hearts by overexpressing a Kir2.1-dominant-negative construct (Kir2.1AAA) via adenoviral mediated gene therapy [22]. This construct allowed ventricular cardiomyocytes to depolarize spontaneously by suppression of the I_K1_ current. Like the β_2_ adrenergic receptor approach, suppression of the I_K1_ current did not create phenotypic pacemaker-like cells but rather attempted to manipulate a single ionic channel, leaving the ventricular myocytes structurally and genetically unaltered. One important concern that was raised because of this study was that downregulation of I_K1_ removed an important determinant of repolarization, leading to prolonged repolarization. This could then result in excessive dispersion of repolarization leading to increased risks of arrhythmia [98]. Follow-up studies demonstrated that overexpression of KIR2.1AAA affected the resting membrane potential resulting in spontaneous depolarizations, but also led to prolongation of action potential duration when less-intensely expressed in the heart [99].

These studies did not characterize the effects of changes to I_K1_ in the myocardium. Hence, stringent studies should be performed to characterize the effects in preclinical models, including large animals with heart rates similar to humans, to rule out potential pro-arrhythmic effects of I_K1_ modulation.

#### 5.2.2. HCN Overexpression

Pacing cells are unique in that they have a slow depolarizing phase, rendering them spontaneously active [100]. This is centered around the function of the HCN channels which increase the inward currents and generate the I_f_ current during the hyperpolarization phase of the action potential. HCN1, 2 and 4 have been studied. HCN4 is the dominant form found in the SAN, HCN2 is the dominant form found in the conducting system and HCN1 is also found in the SAN but is less optimal for pacemaker targeting [101,102,103,104]. HCN2 has been the target of overexpression because of its favorable activation kinetics over the other HCN channels.

One of the first attempts at targeting the HCN channels for functional reengineering to create pacemaker cells involved the overexpression of HCN2 in a canine model [98]. Adenoviral constructs expressing the mouse HCN2 gene were delivered via epicardial injection to the root of the left atrial appendage. Four days after delivery, spontaneous beats were detected originating at the injection site. The heart rate was enhanced with catecholamines and suppressed by left vagal stimulation, showing autonomic responsiveness. Cells examined from the site of injection showed increased expression of HCN2 channels and currents, confirming effectiveness of the approach.

Follow up studies injected the same HCN2 construct into the left bundle branch of canines in sinus rhythm, subjected to transient atrioventricular block and vagal stimulation [76]. Biological pacemaker function compared well to the electronic units implanted into the controls. The major downside however was the ideal basal and maximal rates were 30–40 bpm and 60–80 bpm slower, respectively.

Improvement is clearly needed for HCN channel overexpression. This has been sought by designing mutant or chimeric HCN constructs that have more-positive activation, enhanced responsiveness or using combined therapies [105,106]. The HCN approach is potentially less pro-arrhythmic compared to I_K1_ suppression as it incorporates the endogenous pacemaker channel gene, which selectively activates only during diastole.

### 5.3. Combined Gene-Cell Approaches

To harness the strengths of stem cells and gene therapy for biological pacemaker development, combined gene-cell delivery platforms have been trialed. This involved overexpressing pacemaker genes in cells before transplantation to increase their ability to form junctions with adjacent cells and to enhance their automaticity.

The most successful preclinical work to date involved injecting human mesenchymal stem cells (hMSC) overexpressing HCN2 into the canine hearts with complete heart block [107,108]. Animals injected with the engineered hMSCs showed biological pacemaker activity with rates of 50–60 bpm and no evidence of cellular or humoral rejection [107]. Advantages of this approach include avoiding the use of viral vectors and immunosuppression. However, fairly low heart rates were achieved as compared to baseline rates. Concerns still exist surrounding the potential migration and further differentiation of hMSCs once delivered to the heart and the efficiency and duration of cell engraftment is still not ideal [109].

Another combined approach used polyethylene glycol to fuse engineered syngeneic fibroblasts overexpressing HCN1, injected into guinea pig hearts [110]. The study documented the formation of fibroblast–myocyte connections, biological pacemaker activity originating at the injection site and β adrenergic responsiveness. Although this alternative represents a non-viral, non-stem-cell-based approach, the major concern includes using polyethylene glycol in a clinical setting and optimizing minimally invasive delivery systems for cell-fusion biological pacemakers.

### 5.4. Somatic Reprogramming to an Induced Pacemaker-Like Phenotype

Advancement in the understanding of how the SAN forms during embryonic development has led to increased recent efforts to develop a biological pacemaker. The new approach now focuses on reactivating developmental pathways to reprogram adult cardiomyocytes into pacemaker like cells. Unlike the stem cell, receptor and channel-based gene therapy approaches, somatic reprogramming involving the overexpression of transcription factors has shown tremendous potential in creating faithful replicas of pacemaker cells [2,75].

The developmental biology of the SAN provides the most relevant information when looking at which transcription factors would provide the most successful outcomes. Previous research highlighted that the functions of the transcription factors Shox2, Tbx3, and Tbx18, along with canonical Wnt signaling were most relevant in the embryonic specification and maturation of the SAN [111,112,113]. This knowledge led to the overexpression of the gene coding for the human embryonic transcription factor TBX18 in ventricular cardiomyocytes of adult guinea pigs in a bradycardic disease model [2]. Transduction of TBX18 induced reprogramming of ventricular cardiomyocytes into SAN cells which resembled endogenous SAN cells. As opposed to previous approaches, no single determinant of excitability was selectively overexpressed. Rather, the entire gene expression profile was altered resulting in fundamental phenotypic changes to the cells, meaning there were no major differences between the reprogrammed cells or their native counterparts. Furthermore, the in vivo reprogramming by TBX18 created a biological pacemaker rhythm in the guinea pig hearts that corrected the disease model, originating from the injection site and was responsive to chronotropic intervention.

Following on from the small animal model using TBX18 for somatic reprogramming, TBX18 was again delivered using an adenoviral vector via a minimally invasive technique in a swine model of complete heart block [75]. The vector encoding TBX18 was delivered to the His-bundle region by a needle catheter without the need for any invasive surgery. The animals were monitored by implanted continuous ECG and activity telemetry devices with the results showing that animals transduced with TBX18 had stable pacemaker activity originating from the injection site while also having a higher heart rate, more diurnal heart rate variation, decreased reliance on the backup electronic pacemaker and an increased physiologic autonomic response to isoproterenol. Moreover, there were no signs of pro-arrhythmia or systemic adverse effects. While these results were the most promising to date, biological pacing in this model peaked around day 7–8 and waned by day 14. The declining heart rate is explained by the host immune response to the adenoviral vector employed [2,57,58,59,60].

## 6. RNA and Small Molecule-Based Therapy Approaches

Recent advances in the use of in vitro transcribed, synthetic mRNA and small molecule biologics have broadened the scope of therapeutic targets for a variety of human diseases because their development and delivery are relatively straightforward [114]. This holds true for dozens of mRNA and small molecule-based therapeutics currently under clinical investigation for the treatment of diseases ranging from genetic disorders, HIV infection, various cancers and cardiovascular diseases [115]. These emerging treatment options demonstrate the unprecedented versatility of these emerging technologies and the wide scope for application. Issues however have hindered the clinical progress of these approaches. mRNA is inherently unstable with the half-life yet to be tested. It is potentially immunogenic, although eliciting a lower immune response compared to adenoviral vector mediated approaches [116,117]. They typically require a delivery vehicle for efficient transport to the targeted cells [115], but myocardial gene transfer efficiency of synthetic mRNA sufficient for reprogramming chamber myocardium to induce pacemaker cells in situ has not been examined. Small molecules can be enzyme inhibitors, receptor ligands, or allosteric modulators, generating unwanted and sometimes serious off target effects [118]. Furthermore, localized delivery of small molecules for extended periods of time may lead to inferior safety profiles compared to gene transfer technologies. These two approaches present another set of alternatives to biological pacemaker development. Their feasibilities however have not been sufficiently explored in this application, requiring extensive investigation for their potential uses.

## 7. Limitations of Gene Therapy Use for Biological Pacemakers

As outlined by this review, there are still many limitations to using gene therapy for the development of biological pacemakers. The use of viral vectors to deliver the necessary genes, presents its own inherent problems. For example, replication deficient adenoviruses only provide transient improvement in pacemaker function largely due to the vector’s ability to induce a strong and rapid inflammatory and immune response that results in vector clearance. Other vectors such as lentivirus are not cardiotropic and do not efficiently transduced the myocardium. There is also a greater risk of insertional mutagenesis.

The appropriate choice of viral vector is the best way to address the major issue of duration of function for biological pacemakers created by gene transfer. The duration of pacemaker function depends on vector-host interactions and ultimately the survival of the vector. This requires vectors that can evade the immune response and confer long term gene expression. To address these vector limitations, AAV vectors present a promising alternative due to several factors. They are highly cardiotropic in nature, they have minimal immunogenicity as compared to other vectors, they allow for longer term gene expression, they have been used in several human clinical trials highlighting their reliability and efficacy as a gene therapy vector and further research is being conducted via directed evolution to create capsids with enhanced cardiotropism and an ability to evade the immune response.

The current approaches mainly rely on the insertion of the pacemaker genes in the proximal conduction system or ventricular myocardium for proof-of-principle studies. There are significant difficulties creating a large animal model of SAN dysfunction and therefore it is unknown if these types of biological pacemakers can drive atrial pacing in the context of SAN disease. This requires further evaluation with comparison of effects of vector injection at different sites around the heart including atrial sites near the native SAN.

Furthermore, the setting in which a biological pacemaker will be used needs to be further examined. The delay between gene delivery and biological function remains limited by the time it takes for the vector to deliver the gene and then begin to function. Hence clinical scenarios where biological pacemaking is used as an adjunct to conventional therapy would be the most suitable entry point into clinical use.

Finally, the autonomic responsiveness of biological pacemakers, the ideal site for implantation, the extent of recovery of pacemaker activity and the ideal construct for gene delivery remain unanswered questions.

## 8. Conclusions

The development of biological pacing via gene therapy has come a long way, yet much remains to be understood if we are to create a therapy that can be given with appropriate safety and predictable efficacy for large populations of individuals. 

Electronic pacemakers are effective therapies, and continued advances in device technology will raise the bar for biological pacemaker therapy. However, advances in embryonic pacemaker development, molecular biology techniques and gene therapy applications provide new opportunities for biological pacemakers that may outperform electronic devices in the future. Treating SAN dysfunction may be feasible in the future, but more work is required to develop clinically relevant animal models. 

More comprehensive testing of biological pacemakers in translational large animal models and Phase I clinical trials will almost certainly employ a tandem approach, with electronic pacemakers providing a safety net. However, eventually, it is envisioned that biological pacemaker will not only confers stability that outlasts that of an electronic pacemaker but will also be individually tailored to the pathophysiology of each patient, responding to their own innate autonomic system. This will all need to be accomplished without any increased risk of rejection, neoplasia, or arrhythmias. Though many important hurdles to biologic therapies exist, given the exciting advances and increasing pace of innovation, it is optimistic that translation of this technology is on the horizon.

## Figures and Tables

**Table 1:** Plasmids and Viral Vectors for Cardiovascular Gene Therapy Applications [69].

Vector	Plasmid	AAV	Lentivirus	Adenovirus
Maximum titre (particles per mL)	N/A	Up to 10^13^	Up to 10^9^	Up to 10^13^
Genome/Size (Kb)	DNA	ssDNA	ssRNA	dsDNA
Insert capacity	15 kB	4.8 kB	10 kB	7 to 30 kB
Integration	No	No	Yes (Random)	No
Length of transgene expression	Up to 2 Months	Long Term	Long Term	Up to 2 Weeks
Immunogenicity	Minimally Immunogenic	Minimally Immunogenic	Minimally Immunogenic	Cytotoxic and Immunogenic
Limited by neutralizing antibodies	No	Yes	No	No
Target cells	Dividing and Non-dividing cells	Dividing and Non-dividing cells	Dividing and Non-dividing cells	Dividing and Non-dividing cells
Cardiac gene transfer	Low Cardiac Transduction	Cardiotropic AAV Serotypes	Lower Cardiac Transduction	High Cardiac Transduction
Disadvantages	Low Transfection Efficiency	Risk of neutralizing antibodies and T-Cell Responses	Risk of insertional mutagenesis	High antibody and inflammatory response
Clinical trial approval	Yes	Yes	Yes	Yes

## References

[1] Bleeker W.K., Mackaay A.J., Masson-Pévet M., Bouman L.N., Becker A.E. (1980). Functional and morphological organization of the rabbit sinus node. Circ. Res..

[2] Kapoor N., Liang W., Marbán E., Cho H.C. (2012). Direct conversion of quiescent cardiomyocytes to pacemaker cells by expression of Tbx18. Nat. Biotechnol..

[3] Gregoratos G. (2003). Sick Sinus Syndrome. Circulation.

[4] Semelka M., Gera J., Usman S. (2013). Sick sinus syndrome: A review. Am. Fam. Physician.

[5] Jensen P.N., Gronroos N.N., Chen L.Y., Folsom A.R., deFilippi C., Heckbert S.R., Alonso A. (2014). Incidence of and risk factors for sick sinus syndrome in the general population. J. Am. Coll. Cardiol..

[6] Adan V., Crown L.A. (2003). Diagnosis and treatment of sick sinus syndrome. Am. Fam. Physician.

[7] Bradshaw P.J., Stobie P., Knuiman M.W., Briffa T.G., Hobbs M.S. (2014). Trends in the incidence and prevalence of cardiac pacemaker insertions in an ageing population. Open Heart.

[8] John R.M., Kumar S. (2016). Sinus Node and Atrial Arrhythmias. Circulation.

[9] Keller K.B., Lemberg L. (2006). The Sick Sinus Syndrome. Am. J. Crit. Care.

[10] Cronin B., Essandoh M.K. (2017). Update on Cardiovascular Implantable Electronic Devices for Anesthesiologists. J. Cardiothorac. Vasc. Anesth..

[11] Mulpuru S.K., Madhavan M., McLeod C.J., Cha Y.-M., Friedman P.A. (2017). Cardiac Pacemakers: Function, Troubleshooting, and Management: Part 1 of a 2-Part Series. J. Am. Coll. Cardiol..

[12] Madhavan M., Mulpuru S.K., McLeod C.J., Cha Y.-M., Friedman P.A. (2017). Advances and Future Directions in Cardiac Pacemakers: Part 2 of a 2-Part Series. J. Am. Coll. Cardiol..

[13] Cingolani E., Goldhaber J.I., Marban E. (2018). Next-generation pacemakers: From small devices to biological pacemakers. Nat. Rev. Cardiol..

[14] Mond H.G., Freitag G. (2014). The cardiac implantable electronic device power source: Evolution and revolution. Pacing Clin. Electrophysiol..

[15] Gillis A.M., Purerfellner H., Israel C.W., Sunthorn H., Kacet S., Anelli-Monti M., Tang F., Young M., Boriani G. (2006). Reducing unnecessary right ventricular pacing with the managed ventricular pacing mode in patients with sinus node disease and AV block. Pacing Clin. Electrophysiol..

[16] Banaszewski M., Stępińska J. (2012). Right heart perforation by pacemaker leads. Arch. Med. Sci..

[17] Khan M.N., Joseph G., Khaykin Y., Ziada K.M., Wilkoff B.L. (2005). Delayed Lead Perforation: A Disturbing Trend. Pacing Clin. Electrophysiol..

[18] DiFrancesco D. (2010). The role of the funny current in pacemaker activity. Circ. Res..

[19] DiFrancesco D., Borer J.S. (2007). The funny current: Cellular basis for the control of heart rate. Drugs.

[20] Dobrzynski H., Boyett M.R., Anderson R.H. (2007). New insights into pacemaker activity: Promoting understanding of sick sinus syndrome. Circulation.

[21] Baruscotti M., Bucchi A., Difrancesco D. (2005). Physiology and pharmacology of the cardiac pacemaker (“funny”) current. Pharmacol. Ther..

[22] Miake J., Marban E., Nuss H.B. (2002). Biological pacemaker created by gene transfer. Nature.

[23] Milanesi R., Baruscotti M., Gnecchi-Ruscone T., DiFrancesco D. (2006). Familial sinus bradycardia associated with a mutation in the cardiac pacemaker channel. N. Engl. J. Med..

[24] Schulze–Bahr E., Neu A., Friederich P., Kaupp U.B., Breithardt G., Pongs O., Isbrandt D. (2003). Pacemaker channel dysfunction in a patient with sinus node disease. J. Clin. Investig..

[25] Ueda K., Nakamura K., Hayashi T., Inagaki N., Takahashi M., Arimura T., Morita H., Higashiuesato Y., Hirano Y., Yasunami M. (2004). Functional characterization of a trafficking-defective HCN4 mutation, D553N, associated with cardiac arrhythmia. J. Biol. Chem..

[26] Saito Y., Nakamura K., Yoshida M., Sugiyama H., Ohe T., Kurokawa J., Furukawa T., Takano M., Nagase S., Morita H. (2015). Enhancement of Spontaneous Activity by HCN4 Overexpression in Mouse Embryonic Stem Cell-Derived Cardiomyocytes—A Possible Biological Pacemaker. PLoS ONE.

[27] Saito Y., Nakamura K., Yoshida M., Sugiyama H., Takano M., Nagase S., Morita H., Kusano K.F., Ito H. (2018). HCN4-Overexpressing Mouse Embryonic Stem Cell-Derived Cardiomyocytes Generate a New Rapid Rhythm in Rats with Bradycardia. Int. Heart J..

[28] Vaidyanathan R., Markandeya Y.S., Kamp T.J., Makielski J.C., January C.T., Eckhardt L.L. (2016). IK1-enhanced human-induced pluripotent stem cell-derived cardiomyocytes: An improved cardiomyocyte model to investigate inherited arrhythmia syndromes. Am. J. Physiol. Heart. Circ. Physiol..

[29] Tse H.-F., Siu C.-W., Chan Y.-C., Lau Y.-M., Lau C.-P., Li R.A. (2009). Synergistic effects of Inward Rectifier (I_K1_) and Pacemaker (I_f_) Currents on the Induction of Bioengineered Cardiac Automaticity. J. Cardiovasc. Electrophysiol..

[30] Xue T., Cho H.C., Akar F.G., Tsang S.Y., Jones S.P., Marban E., Tomaselli G.F., Li R.A. (2005). Functional integration of electrically active cardiac derivatives from genetically engineered human embryonic stem cells with quiescent recipient ventricular cardiomyocytes: Insights into the development of cell-based pacemakers. Circulation.

[31] Ionta V., Liang W., Kim E.H., Rafie R., Giacomello A., Marbán E., Cho H.C. (2015). SHOX2 overexpression favors differentiation of embryonic stem cells into cardiac pacemaker cells, improving biological pacing ability. Stem Cell Rep..

[32] Saito Y., Nakamura K., Ito H. (2018). Cell-based Biological Pacemakers: Progress and Problems. Acta Med. Okayama.

[33] Gepstein L. (2005). Stem cells as biological heart pacemakers. Expert Opin. Biol. Ther..

[34] Ruhparwar A., Tebbenjohanns J., Niehaus M., Mengel M., Irtel T., Kofidis T., Pichlmaier A.M., Haverich A. (2002). Transplanted fetal cardiomyocytes as cardiac pacemaker. Eur. J. Cardiothorac. Surg..

[35] Xu C., Police S., Rao N., Carpenter M.K. (2002). Characterization and enrichment of cardiomyocytes derived from human embryonic stem cells. Circ. Res..

[36] Protze S.I., Liu J., Nussinovitch U., Ohana L., Backx P.H., Gepstein L., Keller G.M. (2017). Sinoatrial node cardiomyocytes derived from human pluripotent cells function as a biological pacemaker. Nat. Biotechnol..

[37] Kehat I., Khimovich L., Caspi O., Gepstein A., Shofti R., Arbel G., Huber I., Satin J., Itskovitz-Eldor J., Gepstein L. (2004). Electromechanical integration of cardiomyocytes derived from human embryonic stem cells. Nat. Biotechnol..

[38] Takahashi K., Yamanaka S. (2006). Induction of Pluripotent Stem Cells from Mouse Embryonic and Adult Fibroblast Cultures by Defined Factors. Cell.

[39] Mandel Y., Weissman A., Schick R., Barad L., Novak A., Meiry G., Goldberg S., Lorber A., Rosen M.R., Itskovitz-Eldor J. (2012). Human Embryonic and Induced Pluripotent Stem Cell–Derived Cardiomyocytes Exhibit Beat Rate Variability and Power-Law Behavior. Circulation.

[40] Chauveau S., Anyukhovskiy Y., Benari M., Naor S., Danilo P., Rahim T., Potapova I., Burke S., Jiang Y.-P., Qiu X. (2016). Keratinocyte-derived cardiomyocytes provide in vivo biological pacemaker function. Arch. Cardiovasc. Dis. Suppl..

[41] Jung J.J., Husse B., Rimmbach C., Krebs S., Stieber J., Steinhoff G., Dendorfer A., Franz W.-M., David R. (2014). Programming and Isolation of Highly Pure Physiologically and Pharmacologically Functional Sinus-Nodal Bodies from Pluripotent Stem Cells. Stem Cell Rep..

[42] Chauveau S., Anyukhovsky E.P., Ben-Ari M., Naor S., Jiang Y.P., Danilo P., Rahim T., Burke S., Qiu X., Potapova I.A. (2017). Induced Pluripotent Stem Cell-Derived Cardiomyocytes Provide in vivo Biological Pacemaker Function. Circ. Arrhythm. Electrophysiol..

[43] Masson-Pevet M.A., Bleeker W.K., Besselsen E., Treytel B.W., Jongsma H.J., Bouman L.N. (1984). Pacemaker cell types in the rabbit sinus node: A correlative ultrastructural and electrophysiological study. J. Mol. Cell. Cardiol..

[44] Boyett M.R., Honjo H., Kodama I. (2000). The sinoatrial node, a heterogeneous pacemaker structure. Cardiovasc. Res..

[45] Dobrzynski H., Li J., Tellez J., Greener I.D., Nikolski V.P., Wright S.E., Parson S.H., Jones S.A., Lancaster M.K., Yamamoto M. (2005). Computer three-dimensional reconstruction of the sinoatrial node. Circulation.

[46] Koivumäki J.T., Naumenko N., Tuomainen T., Takalo J., Oksanen M., Puttonen K.A., Lehtonen Š., Kuusisto J., Laakso M., Koistinaho J. (2018). Structural Immaturity of Human iPSC-Derived Cardiomyocytes: In Silico Investigation of Effects on Function and Disease Modeling. Front. Physiol..

[47] Liu Z., Tang Y., Lü S., Zhou J., Du Z., Duan C., Li Z., Wang C. (2013). The tumourigenicity of iPS cells and their differentiated derivates. J. Cell. Mol. Med..

[48] Narsinh K., Narsinh K.H., Wu J.C. (2011). Derivation of human induced pluripotent stem cells for cardiovascular disease modeling. Circ. Res..

[49] Lugenbiel P., Thomas D., Kelemen K., Trappe K., Bikou O., Schweizer P.A., Voss F., Becker R., Katus H.A., Bauer A. (2012). Genetic suppression of Galphas protein provides rate control in atrial fibrillation. Basic Res. Cardiol..

[50] Trappe K., Thomas D., Bikou O., Kelemen K., Lugenbiel P., Voss F., Becker R., Katus H.A., Bauer A. (2013). Suppression of persistent atrial fibrillation by genetic knockdown of caspase 3: A pre-clinical pilot study. Eur. Heart J..

[51] Maeder M.L., Gersbach C.A. (2016). Genome-editing Technologies for Gene and Cell Therapy. Mol. Ther..

[52] French B.A., Mazur W., Geske R.S., Bolli R. (1994). Direct in vivo gene transfer into porcine myocardium using replication-deficient adenoviral vectors. Circulation.

[53] Kizana E., Alexander I.E. (2003). Cardiac gene therapy: Therapeutic potential and current progress. Curr. Gene. Ther..

[54] Rincon M.Y., VandenDriessche T., Chuah M.K. (2015). Gene therapy for cardiovascular disease: Advances in vector development, targeting, and delivery for clinical translation. Cardiovasc. Res..

[55] Schmidt-Wolf G.D., Schmidt-Wolf I.G.H. (2003). Non-viral and hybrid vectors in human gene therapy: An update. Trends Mol. Med..

[56] Liu Z., Donahue J.K. (2014). The Use of Gene Therapy for Ablation of Atrial Fibrillation. Arrhythm. Electrophysiol. Rev..

[57] Liu Q., Zaiss A.K., Colarusso P., Patel K., Haljan G., Wickham T.J., Muruve D.A. (2003). The role of capsid-endothelial interactions in the innate immune response to adenovirus vectors. Hum. Gene Ther..

[58] Muruve D.A. (2004). The innate immune response to adenovirus vectors. Hum. Gene Ther..

[59] Lusky M., Christ M., Rittner K., Dieterle A., Dreyer D., Mourot B., Schultz H., Stoeckel F., Pavirani A., Mehtali M. (1998). In vitro and in vivo biology of recombinant adenovirus vectors with E1, E1/E2A, or E1/E4 deleted. J. Virol..

[60] Gorziglia M.I., Kadan M.J., Yei S., Lim J., Lee G.M., Luthra R., Trapnell B.C. (1996). Elimination of both E1 and E2 from adenovirus vectors further improves prospects for in vivo human gene therapy. J. Virol..

[61] Zhao J., Pettigrew G.J., Thomas J., Vandenberg J.I., Delriviere L., Bolton E.M., Carmichael A., Martin J.L., Marber M.S., Lever A.M.L. (2002). Lentiviral vectors for delivery of genes into neonatal and adult ventricular cardiac myocytes in vitro and in vivo. Basic Res. Cardiol..

[62] Williams P.D., Ranjzad P., Kakar S.J., Kingston P.A. (2010). Development of Viral Vectors for Use in Cardiovascular Gene Therapy. Viruses.

[63] Hacein-Bey-Abina S., Von Kalle C., Schmidt M., McCormack M.P., Wulffraat N., Leboulch P., Lim A., Osborne C.S., Pawliuk R., Morillon E. (2003). LMO2-associated clonal T cell proliferation in two patients after gene therapy for SCID-X1. Science.

[64] Howe S.J., Mansour M.R., Schwarzwaelder K., Bartholomae C., Hubank M., Kempski H., Brugman M.H., Pike-Overzet K., Chatters S.J., de Ridder D. (2008). Insertional mutagenesis combined with acquired somatic mutations causes leukemogenesis following gene therapy of SCID-X1 patients. J. Clin. Investig..

[65] Chamberlain K., Riyad J.M., Weber T. (2017). Cardiac gene therapy with adeno-associated virus-based vectors. Curr. Opin. Cardiol..

[66] Zacchigna S., Zentilin L., Giacca M. (2014). Adeno-associated virus vectors as therapeutic and investigational tools in the cardiovascular system. Circ. Res..

[67] Pacak C.A., Byrne B.J. (2011). AAV vectors for cardiac gene transfer: Experimental tools and clinical opportunities. Mol. Ther..

[68] Wu Z., Asokan A., Samulski R.J. (2006). Adeno-associated virus serotypes: Vector toolkit for human gene therapy. Mol. Ther..

[69] Farraha M., Chong J.J., Kizana E. (2016). Therapeutic Prospects of Gene Therapy for Atrial Fibrillation. Heart Lung Circ..

[70] Asokan A., Conway J.C., Phillips J.L., Li C., Hegge J., Sinnott R., Yadav S., DiPrimio N., Nam H.J., Agbandje-McKenna M. (2010). Reengineering a receptor footprint of adeno-associated virus enables selective and systemic gene transfer to muscle. Nat. Biotechnol..

[71] Kotterman M.A., Schaffer D.V. (2014). Engineering adeno-associated viruses for clinical gene therapy. Nat. Rev. Genet..

[72] Li W., Zhang L., Johnson J.S., Zhijian W., Grieger J.C., Ping-Jie X., Drouin L.M., Agbandje-McKenna M., Pickles R.J., Samulski R.J. (2009). Generation of Novel AAV Variants by Directed Evolution for Improved CFTR Delivery to Human Ciliated Airway Epithelium. Mol. Ther..

[73] Paulk N.K., Pekrun K., Lisowski L., Zhang Y., Chu K., Kay M.A. (2015). Directed Evolution of Improved AAV Capsids for the Ideal Human Liver Vector, 2013; Can Human Liver Tropism and Human Immune Evasion Be Achieved?. Mol. Ther..

[74] Yang L., Li J., Xiao X. (2011). Directed evolution of adeno-associated virus (AAV) as vector for muscle gene therapy. Methods Mol. Biol..

[75] Hu Y.F., Dawkins J.F., Cho H.C., Marban E., Cingolani E. (2014). Biological pacemaker created by minimally invasive somatic reprogramming in pigs with complete heart block. Sci. Transl. Med..

[76] Plotnikov A.N., Sosunov E.A., Qu J., Shlapakova I.N., Anyukhovsky E.P., Liu L., Janse M.J., Brink P.R., Cohen I.S., Robinson R.B. (2004). Biological pacemaker implanted in canine left bundle branch provides ventricular escape rhythms that have physiologically acceptable rates. Circulation.

[77] Kikuchi K., McDonald A.D., Sasano T., Donahue J.K. (2005). Targeted modification of atrial electrophysiology by homogeneous transmural atrial gene transfer. Circulation.

[78] Igarashi T., Finet J.E., Takeuchi A., Fujino Y., Strom M., Greener I.D., Rosenbaum D.S., Donahue J.K. (2012). Connexin gene transfer preserves conduction velocity and prevents atrial fibrillation. Circulation.

[79] Donahue J.K. (2016). Biological Therapies for Atrial Fibrillation: Ready for Prime Time?. J. Cardiovasc. Pharmacol..

[80] Boink G.J., Duan L., Nearing B.D., Shlapakova I.N., Sosunov E.A., Anyukhovsky E.P., Bobkov E., Kryukova Y., Ozgen N., Danilo P. (2013). HCN2/SkM1 gene transfer into canine left bundle branch induces stable, autonomically responsive biological pacing at physiological heart rates. J. Am. Coll. Cardiol..

[81] Aistrup G.L., Cokic I., Ng J., Gordon D., Koduri H., Browne S., Arapi D., Segon Y., Goldstein J., Angulo A. (2011). Targeted nonviral gene-based inhibition of Galpha(i/o)-mediated vagal signaling in the posterior left atrium decreases vagal-induced atrial fibrillation. Heart Rhythm.

[82] Bikou O., Thomas D., Trappe K., Lugenbiel P., Kelemen K., Koch M., Soucek R., Voss F., Becker R., Katus H.A. (2011). Connexin 43 gene therapy prevents persistent atrial fibrillation in a porcine model. Cardiovasc. Res..

[83] Li J.J., Ueno H., Pan Y., Tomita H., Yamamoto H., Kanegae Y., Saito I., Takeshita A. (1995). Percutaneous transluminal gene transfer into canine myocardium in vivo by replication-defective adenovirus. Cardiovasc. Res..

[84] Hargrave B., Downey H., Strange R., Murray L., Cinnamond C., Lundberg C., Israel A., Chen Y.J., Marshall W., Heller R. (2013). Electroporation-mediated gene transfer directly to the swine heart. Gene. Ther..

[85] Donahue J.K., Heldman A.W., Fraser H., McDonald A.D., Miller J.M., Rade J.J., Eschenhagen T., Marban E. (2000). Focal modification of electrical conduction in the heart by viral gene transfer. Nat. Med..

[86] Donahue J.K., Kikkawa K., Thomas A.D., Marban E., Lawrence J.H. (1998). Acceleration of widespread adenoviral gene transfer to intact rabbit hearts by coronary perfusion with low calcium and serotonin. Gene Ther..

[87] Karakikes I., Hadri L., Rapti K., Ladage D., Ishikawa K., Tilemann L., Yi G.H., Morel C., Gwathmey J.K., Zsebo K. (2012). Concomitant intravenous nitroglycerin with intracoronary delivery of AAV1.SERCA2a enhances gene transfer in porcine hearts. Mol. Ther..

[88] Roth D.M., Lai N.C., Gao M.H., Fine S., McKirnan M.D., Roth D.A., Hammond H.K. (2004). Nitroprusside increases gene transfer associated with intracoronary delivery of adenovirus. Hum. Gene Ther..

[89] Nagata K., Marban E., Lawrence J.H., Donahue J.K. (2001). Phosphodiesterase inhibitor-mediated potentiation of adenovirus delivery to myocardium. J. Mol. Cell. Cardiol..

[90] Sasano T., Kikuchi K., McDonald A.D., Lai S., Donahue J.K. (2007). Targeted High-Efficiency, Homogeneous Myocardial Gene Transfer. J. Mol. Cell. Cardiol..

[91] Donahue J.K., Kikkawa K., Johns D.C., Marban E., Lawrence J.H. (1997). Ultrarapid, highly efficient viral gene transfer to the heart. Proc. Natl. Acad. Sci. USA.

[92] Lugenbiel P., Bauer A., Kelemen K., Schweizer P.A., Becker R., Katus H.A., Thomas D. (2012). Biological Heart Rate Reduction Through Genetic Suppression of Gαs Protein in the Sinoatrial Node. J. Am. Heart Assoc..

[93] Edelberg J.M., Aird W.C., Rosenberg R.D. (1998). Enhancement of murine cardiac chronotropy by the molecular transfer of the human beta2 adrenergic receptor Cdna. J. Clin. Investig..

[94] Rodefeld M.D., Beau S.L., Schuessler R.B., Boineau J.P., Saffitz J.E. (1996). Beta-adrenergic and muscarinic cholinergic receptor densities in the human sinoatrial node: Identification of a high beta 2-adrenergic receptor density. J. Cardiovasc. Electrophysiol..

[95] Lakatta E.G., DiFrancesco D. (2009). What keeps us ticking: A funny current, a calcium clock, or both?. J. Mol. Cell. Cardiol..

[96] Piron J., Quang K.L., Briec F., Amirault J.C., Leoni A.L., Desigaux L., Escande D., Pitard B., Charpentier F. (2008). Biological pacemaker engineered by nonviral gene transfer in a mouse model of complete atrioventricular block. Mol. Ther..

[97] Edelberg J., Huang D., Josephson M., Rosenberg R. (2001). Molecular enhancement of porcine cardiac chronotropy. Heart.

[98] Qu J., Plotnikov A.N., Danilo P., Shlapakova I., Cohen I.S., Robinson R.B., Rosen M.R. (2003). Expression and function of a biological pacemaker in canine heart. Circulation.

[99] Miake J., Marban E., Nuss H.B. (2003). Functional role of inward rectifier current in heart probed by Kir2.1 overexpression and dominant-negative suppression. J. Clin. Investig..

[100] DiFrancesco D. (1993). Pacemaker mechanisms in cardiac tissue. Annu. Rev. Physiol..

[101] Biel M., Schneider A., Wahl C. (2002). Cardiac HCN channels: Structure, function, and modulation. Trends Cardiovasc. Med..

[102] Joung B., Tang L., Maruyama M., Han S., Chen Z., Stucky M., Jones L.R., Fishbein M.C., Weiss J.N., Chen P.S. (2009). Intracellular calcium dynamics and acceleration of sinus rhythm by beta-adrenergic stimulation. Circulation.

[103] Cohen I.S., Robinson R.B. (2006). Pacemaker current and automatic rhythms: Toward a molecular understanding. Basis and Treatment of Cardiac Arrhythmias.

[104] Moosmang S., Stieber J., Zong X., Biel M., Hofmann F., Ludwig A. (2001). Cellular expression and functional characterization of four hyperpolarization-activated pacemaker channels in cardiac and neuronal tissues. Eur. J. Biochem..

[105] Bucchi A., Plotnikov A.N., Shlapakova I., Danilo P., Kryukova Y., Qu J., Lu Z., Liu H., Pan Z., Potapova I. (2006). Wild-type and mutant HCN channels in a tandem biological-electronic cardiac pacemaker. Circulation.

[106] Tse H.F., Xue T., Lau C.P., Siu C.W., Wang K., Zhang Q.Y., Tomaselli G.F., Akar F.G., Li R.A. (2006). Bioartificial sinus node constructed via in vivo gene transfer of an engineered pacemaker HCN Channel reduces the dependence on electronic pacemaker in a sick-sinus syndrome model. Circulation.

[107] Plotnikov A.N., Shlapakova I., Szabolcs M.J., Danilo P., Lorell B.H., Potapova I.A., Lu Z., Rosen A.B., Mathias R.T., Brink P.R. (2007). Xenografted adult human mesenchymal stem cells provide a platform for sustained biological pacemaker function in canine heart. Circulation.

[108] Potapova I., Plotnikov A., Lu Z., Danilo P., Valiunas V., Qu J., Doronin S., Zuckerman J., Shlapakova I.N., Gao J. (2004). Human mesenchymal stem cells as a gene delivery system to create cardiac pacemakers. Circ. Res..

[109] Liechty K.W., MacKenzie T.C., Shaaban A.F., Radu A., Moseley A.M., Deans R., Marshak D.R., Flake A.W. (2000). Human mesenchymal stem cells engraft and demonstrate site-specific differentiation after in utero transplantation in sheep. Nat. Med..

[110] Cho H.C., Kashiwakura Y., Marban E. (2007). Creation of a biological pacemaker by cell fusion. Circ. Res..

[111] Cai C.L., Martin J.C., Sun Y., Cui L., Wang L., Ouyang K., Yang L., Bu L., Liang X., Zhang X. (2008). A myocardial lineage derives from Tbx18 epicardial cells. Nature.

[112] Liu H., Espinoza-Lewis R.A., Chen C., Hu X., Zhang Y., Chen Y. (2012). The role of Shox2 in SAN development and function. Pediatr. Cardiol..

[113] McNally E.M., Svensson E.C. (2009). Setting the pace: Tbx3 and Tbx18 in cardiac conduction system development. Circ. Res..

[114] Peer D., Lieberman J. (2011). Special delivery: Targeted therapy with small RNAs. Gene Ther..

[115] Burnett J.C., Rossi J.J. (2012). RNA-based Therapeutics: Current Progress and Future Prospects. Chem. Biol..

[116] Zangi L., Lui K.O., von Gise A., Ma Q., Ebina W., Ptaszek L.M., Spater D., Xu H., Tabebordbar M., Gorbatov R. (2013). Modified mRNA directs the fate of heart progenitor cells and induces vascular regeneration after myocardial infarction. Nat. Biotechnol..

[117] Kauffman K.J., Mir F.F., Jhunjhunwala S., Kaczmarek J.C., Hurtado J.E., Yang J.H., Webber M.J., Kowalski P.S., Heartlein M.W., DeRosa F. (2016). Efficacy and Immunogenicity of Unmodified and Pseudouridine-Modified mRNA Delivered Systemically with Lipid Nanoparticles in vivo. Biomaterials.

[118] Gurevich E.V., Gurevich V.V. (2014). Therapeutic Potential of Small Molecules and Engineered Proteins. Handb. Exp. Pharmacol..

